# Prognostic Signature and Tumor Immune Landscape of N7-Methylguanosine-Related lncRNAs in Hepatocellular Carcinoma

**DOI:** 10.3389/fgene.2022.906496

**Published:** 2022-07-22

**Authors:** Wei Wei, Chao Liu, Meng Wang, Wei Jiang, Caihong Wang, Shuqun Zhang

**Affiliations:** ^1^ Department of Oncology, The Second Affiliated Hospital of Xi’an Jiaotong University, Xi’an, China; ^2^ Department of Vascular Surgery, The First Affiliated Hospital of Xi’an Jiaotong University, Xi’an, China; ^3^ Department of Pathology, The Second Affiliated Hospital of Xi’an Jiaotong University, Xi’an, China

**Keywords:** m7G methylation, lncRNA, LIHC, prognostic model, immune response

## Abstract

Despite great advances in the treatment of liver hepatocellular carcinoma (LIHC), such as immunotherapy, the prognosis remains extremely poor, and there is an urgent need to develop novel diagnostic and prognostic markers. Recently, RNA methylation-related long non-coding RNAs (lncRNAs) have been demonstrated to be novel potential biomarkers for tumor diagnosis and prognosis as well as immunotherapy response, such as N6-methyladenine (m6A) and 5-methylcytosine (m5C). N7-Methylguanosine (m7G) is a widespread RNA modification in eukaryotes, but the relationship between m7G-related lncRNAs and prognosis of LIHC patients as well as tumor immunotherapy response is still unknown. In this study, based on the LIHC patients’ clinical and transcriptomic data from TCGA database, a total of 992 m7G-related lncRNAs that co-expressed with 22 m7G regulatory genes were identified using Pearson correlation analysis. Univariate regression analysis was used to screen prognostic m7G-related lncRNAs, and the least absolute shrinkage and selection operator (LASSO) and multivariate Cox regression were applied to construct a 9-m7G-related-lncRNA risk model. The m7G-related lncRNA risk model was validated to exhibit good prognostic performance through Kaplan–Meier analysis and ROC analysis. Together with the clinicopathological features, the m7G-related lncRNA risk score was found to be an independent prognostic factor for LIHC. Furthermore, the high-risk group of LIHC patients was unveiled to have a higher tumor mutation burden (TMB), and their tumor microenvironment was more prone to the immunosuppressive state and exhibited a lower response rate to immunotherapy. In addition, 47 anti-cancer drugs were identified to exhibit a difference in drug sensitivity between the high-risk and low-risk groups. Taken together, the m7G-related lncRNA risk model might display potential value in predicting prognosis, immunotherapy response, and drug sensitivity in LIHC patients.

## Introduction

Liver hepatocellular carcinoma (LIHC), accounting for approximately 90% cases of the liver non-metastatic tumors, is a global health problem with increasing incidence and mortality (Llovet et al., 2021). For early-stage LIHC, hepatic resection and local ablative therapy are the standard clinical treatment manners (Kamarajah et al., 2021). Owing to insidious onset, rapid progression, and difficulties in early diagnosis, the majority of LIHC patients are first diagnosed at an advanced stage, at which point therapeutic options are limited and ineffective. In the past decade, molecular targeted therapy has become the mainstay of the treatment in advanced-stage LIHC, mainly including sorafenib and lenvatinib as first-line therapy, and regorafenib and ramucirumab, as well as cabozantinib as second-line therapy (Huang A. et al., 2020). Excitingly, immunotherapies, especially immune checkpoint inhibitors (ICIs), have rapidly developed in the past few years and have been proven to be an effective treatment for LIHC with long-term survival (Llovet et al., 2022). At present, nivolumab and pembrolizumab, two anti-PD-1 antibodies, have been used in the clinical treatment of LIHC (Wong et al., 2021). However, LIHC is easy to metastasize and develop drug resistance and has a special tumor immune microenvironment (TIME) (Oura et al., 2021); thus, the current therapeutic effect and prognosis of LIHC patients are still not optimistic, and more novel accurate molecular biomarkers are needed to improve the diagnostic and treatment efficacy of LIHC patients.

Long non-coding RNAs (lncRNAs) are featured as more than 200 nucleotides in length and lack the ability of protein coding. LncRNAs have similar structures to mRNA, including 5′-cap, 3′-poly(A) tail, and promoter structure and can be transcribed by RNA polymerase II (Statello et al., 2021). The number of lncRNAs encoded by human is huge and exceeds 170,000 (Zhao et al., 2021). LncRNAs can modulate gene expression in both cis- and trans-manners at the transcriptional, epigenetic, and post-transcriptional levels and have been demonstrated to be critical regulators of the development and progression of cancer, including LIHC (Gao et al., 2020).

In recent years, epigenetic modifications of lncRNA, such as N6-methyladenine (m6A) and 5-methylcytosine (m5C), have been found to be associated with carcinogenesis and the development of multiple cancers (Wang et al., 2020; Zhang et al., 2021). N7-Methylguanosine (m7G) is a kind of positively charged RNA modification, which is generated by the addition of a methyl group to the N7 atom of guanine (G) by RNA methyltransferase, such as METTL1 (Orellana et al., 2021). M7G modifications are widely present in various RNA molecules including mRNA 5′ cap structure, internal mRNA, transfer RNA (tRNA), rRNA ribosomal RNA (rRNA), and primary microRNA (pri-miRNA), as well as lncRNA, and it has been revealed that m7G can modulate mRNA transcription, mRNA translation, splicing, tRNA stability, nuclear processing, 18 S rRNA maturation, and miRNA biosynthesis (Malbec et al., 2019; Pandolfini et al., 2019; Wang et al., 2022). Similar to m6A and m5C, m7G was recently demonstrated to play fundamental roles in LIHC. For example, METTL1, an m7G methyltransferase, has been linked to advanced tumor stage, vascular invasion, and poor prognosis in LIHC patients and facilitates tumor progression through increasing m7G tRNA modification and promoting translation of target mRNAs (Tian et al., 2019; Chen et al., 2021). Unfortunately, the specific function of m7G-related lncRNAs in the prognosis of LIHC patients remains unclear. Thus, a novel m7G-related lncRNA signature may be helpful in prognostic prediction and treatment response evaluation in LIHC.

In this study, based on the LIHC patients’ clinical and transcriptomic data from TCGA database, a total of 992 m7G-related lncRNAs that co-expressed with 22 m7G regulatory genes were identified using Pearson correlation analysis. Univariate regression analysis was used to screen prognostic m7G-related lncRNAs, and the least absolute shrinkage and selection operator (LASSO) and multivariate Cox regression were applied to construct a 9-m7G-related-lncRNA risk model. The risk model was further verified by Kaplan–Meier analysis and ROC analysis. Furthermore, the roles of the risk model in evaluating tumor mutation burden, immune microenvironment, and immunotherapy response as well as drug sensitivity were explored. In summary, we constructed a 9-m7G-related-lncRNA risk model which may provide promising prognostic value and play essential roles in predicting immune therapy response and chemotherapy sensitivity.

## Materials and Methods

### Data Source and Preprocessing

The transcriptome data, mutation data, and corresponding clinicopathological data of LIHC patients were obtained from TCGA database (https://portal.gdc.cancer.gov/) on 8 February 2022. There were 371 LIHC samples and 50 normal subjects. The expression matrix of these primary samples was normalized in the R package “DESeq2” by variance stabilizing transformation function, and each normalized count was formulated in log2(X +1), where X is each of the normalized counts. Moreover, LIHC patients with missing OS status and times were deleted to reduce bias in statistical analysis.

### Selection of m7G Regulator Genes and m7G-Related lncRNAs

A total of 22 m7G modification-related genes were screened from the published literature works, namely, METTL1 (Ma et al., 2021), WDR4 (Ma et al., 2021), DCP2 (Wurm and Sprangers, 2019), DCPS (Wulf et al., 2019), NUDT2 (Song et al., 2013), NUDT3 (Song et al., 2013), NUDT12 (Song et al., 2013), NUDT15 (Song et al., 2013), NUDT16 (Song et al., 2013), NUDT17 (Song et al., 2013), AGO2 (Kiriakidou et al., 2007), CYFIP1 (Napoli et al., 2008), EIF4E (Napoli et al., 2008), EIF4E2 (Rosettani et al., 2007), EIF4E3 (Osborne et al., 2013), EIF3D (Lee et al., 2016), EIF4A1 (Chu et al., 2020), EIF4G3 (Haghighat and Sonenberg, 1997), GEMIN5 (Bradrick and Gromeier, 2009), LARP1 (Philippe et al., 2018), NCBP1 (Dou et al., 2020), and NCBP2 (Dou et al., 2020). After the annotation of the expression matrix of LIHC samples, the profiles of 22 m7G regulator genes and 13,541 lncRNAs were obtained. Then, a preliminary screening was performed based on the rule that the median expression/variance of each generally changed lncRNA in every sample was 20% higher than the total median expression/variance of all lncRNAs in every patient (Luo et al., 2018), and 4,464 distinctively expressed lncRNAs were screened for further Pearson’s correlation analysis. Finally, 992 m7G-related lncRNAs were identified under the criterion of |correlation coefficient| > 0.3 and *p*-value < 0.01.

### Construction of the Prognostic Risk Model of m7G-Related lncRNAs

The entire LIHC patients were separated into a training set (*n* = 219) and testing set (*n* = 146) randomly, and the m7G-related lncRNA risk model was constructed based on the training set. First of all, prognostic lncRNAs were selected from 992 m7G-related lncRNAs using univariate regression analysis, and then LASSO-penalized Cox regression was further adopted to optimize indicators predicting clinical outcome by using the R package “glmnet.” Finally, a 9-m7G-related-lncRNA prognostic risk model was constructed by multivariate regression analysis. The risk score was computed in the following manner: risk score = coef (lncRNA1) × expr (lncRNA1) + coef (lncRNA2) × expr (lncRNA2) + … + coef (lncRNAn) × expr (lncRNAn). In this formula, the coefficient and corresponding expression value of each lncRNA were calculated, respectively.

### Validation and Evaluation of the Prognostic Risk Model of m7G-Related lncRNAs

The entire LIHC patients and the testing set were applied to validate the efficacy of the 9-m7G-related lncRNA risk model. Based on the median risk score, LIHC patients were categorized into high- and low-risk groups. Principal component analysis (PCA) was conducted to visualize the grouping ability of high-dimensional data in the entire gene set, 22 m7G regulator gene set, 992 m7G-related lncRNAs, and 9 m7G-related lncRNAs expression profiles. The R packages “survival” and “survminer” were adopted to perform KM survival analysis to analyze the differences in the overall survival (OS), disease-specific survival (DSS), disease-free interval (DFI), and progression-free interval (PFI) between the two risk groups. The 1-year, 3-year, and 5-year area under the ROC (AUROC) values were calculated to evaluate the predicting performance of the risk model.

### Independence of the 9-m7G-Related-lncRNA Risk Model in LIHC Patients

We stratified the clinicopathological features of LIHC patients according to age, gender, grade, and stage and analyzed the difference of OS between the two risk groups in the entire set. Moreover, univariate and multivariate Cox regression analyses were calculated to further evaluate the independent prognostic factors in LIHC patients, such as m7G-related lncRNA risk score, age, gender, grade, and stage. The ROC curve of each clinicopathological feature was drawn to show its prognostic value.

### The Predictive Ability of the Nomogram in LIHC Patients

We constructed the nomogram consisting of clinicopathological features (including age, gender, tumor grade, and stage) along with the m7G-related lncRNA risk score to predict the survival status in entire LIHC patients. Moreover, the calibration curve was plotted to evaluate the consistency between the actual and predicted survival of 1-, 3-, and 5-year OS.

### Evaluation of the Tumor Immune Microenvironment Using the Risk Model

KEGG analysis was performed to identify the changed pathways between the two risk groups. KEGG gene sets were downloaded from the MSigDB (https://www.gsea-msigdb.org/gsea/msigdb/), and gene set enrichment analysis (GSEA) of KEGG pathways was implemented using the R package “clusterProfiler.” To evaluate the immune landscape in LIHC patients among different risk groups, we used the R package “GSVA” to perform single-sample GSEA (ssGSEA) to quantify the infiltration level of 28 immune cell types and 13 immune-related functions in each sample.

### Exploring the Immunotherapeutic Treatment Response Targeting the Risk Model

The tumor mutation burden (TMB) was estimated by using the R package “maftools” in both risk groups. The KM survival analysis was performed to analyze the differences in the OS stratified by TMB status and m7G-related lncRNA risk scores in LIHC patients. The tumor immune dysfunction and exclusion (TIDE) score (http://tide.dfci.harvard.edu/) was calculated to evaluate the response to immunotherapy. Furthermore, the expression level of immune checkpoint molecules, such as CD274, PDCD1LG2, LAG3, SIGLEC15, TIGIT, IDO1, CTLA4, and CD276, was compared between the two risk groups in entire LIHC patients.

### Identification of Novel Candidate Compounds for Chemotherapy Based on the Risk Model

To identify novel candidate compounds in LIHC patients for clinical practice, the half-maximal inhibitory concentration (IC_50_) of 138 compounds obtained from the Genomics of Drug Sensitivity in Cancer (GDSC) database was calculated for each LIHC patient by using the R package “pRRophetic,” and the value of IC_50_ of each compound between the two risk groups was compared separately.

## Results

### Identification of m7G-Related lncRNAs in LIHC Patients

**FIGURE 1:**
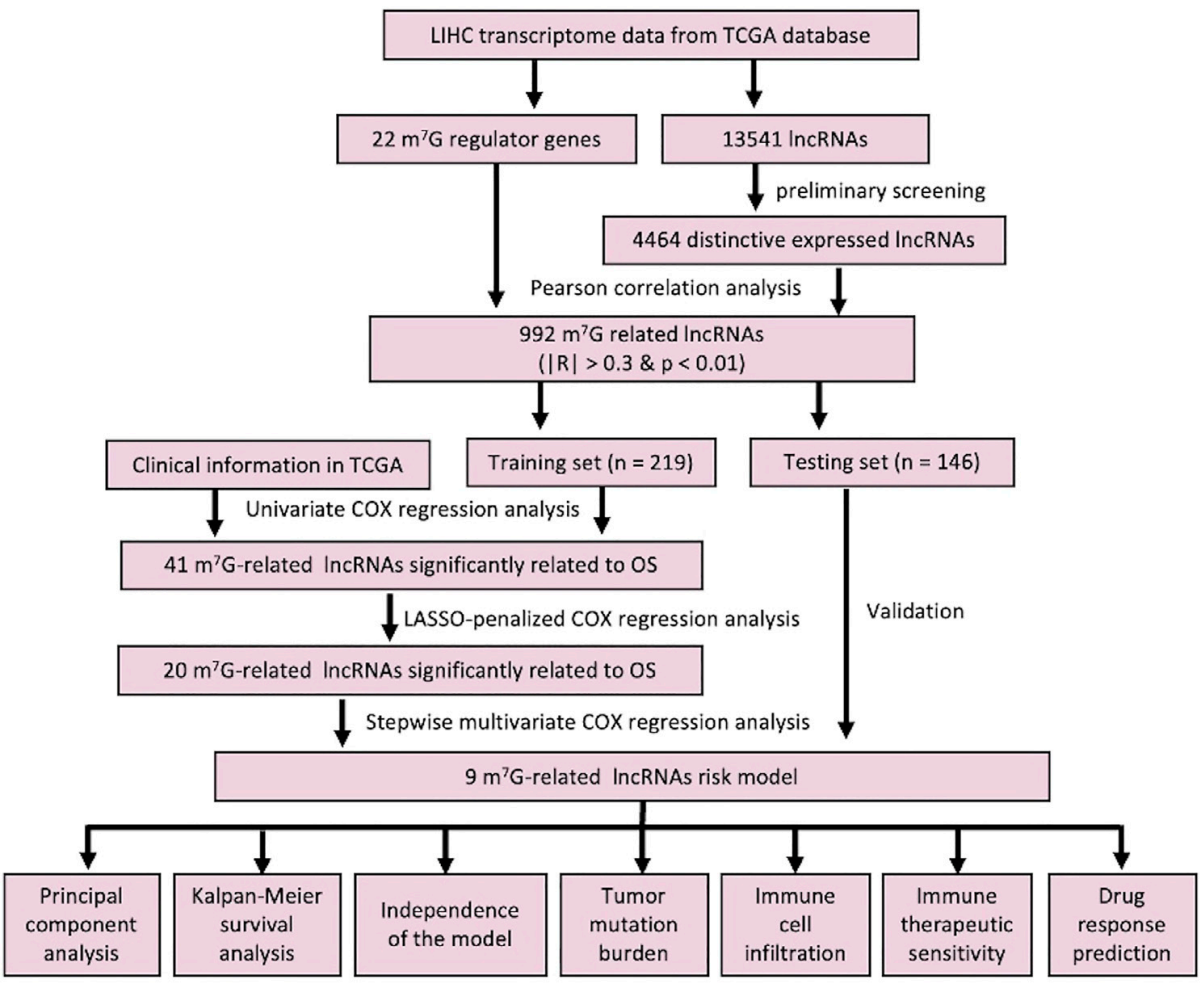
Detailed workflow of this study.

**FIGURE 2:**
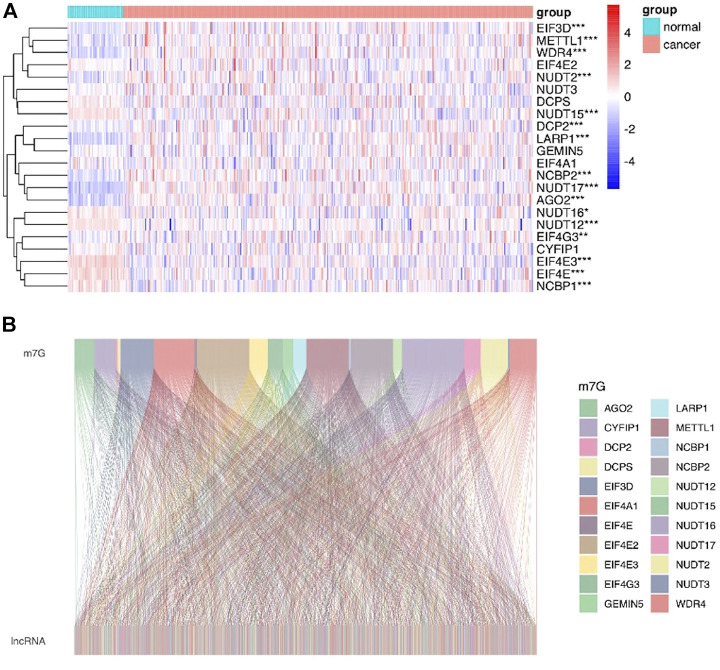
Identification of m7G-related lncRNAs in LIHC patients. **(A)** Heatmap showed the differences in the expression of m7G regulators between LIHC and normal groups. **(B)** Sankey diagram displayed the relationship between 22 m7G genes and 992 m7G-related lncRNAs.

The workflow of this study is described in detail in Figure 1. Initially, we totally screened 22 m7G regulatory genes, including 2 “writers,” 8 “erasers,” and 12 “readers” through literature retrieval, and most of them (16/22) were found to be significantly changed in LIHC (*p* < 0.05) based on TCGA data (Figure 2A). To identify m7G-related lncRNAs in LIHC, expression data of 13,541 lncRNAs in the LIHC cohort was obtained from TCGA database, and 4,464 generally changed lncRNAs with distinctive expression among different LIHC patients were obtained through preliminary screening. Then, we performed correlation analysis and filtered 992 m7G-related lncRNAs under the filter criterion of |correlation coefficient| > 0.3 and *p*-value < 0.01. The network of m7G regulators and corresponding lncRNAs was illustrated using the Sankey diagram (Figure 2B).

### Construction and Validation of a 9-m7G-Related-lncRNA Risk Model for LIHC Patients

**FIGURE 3:**
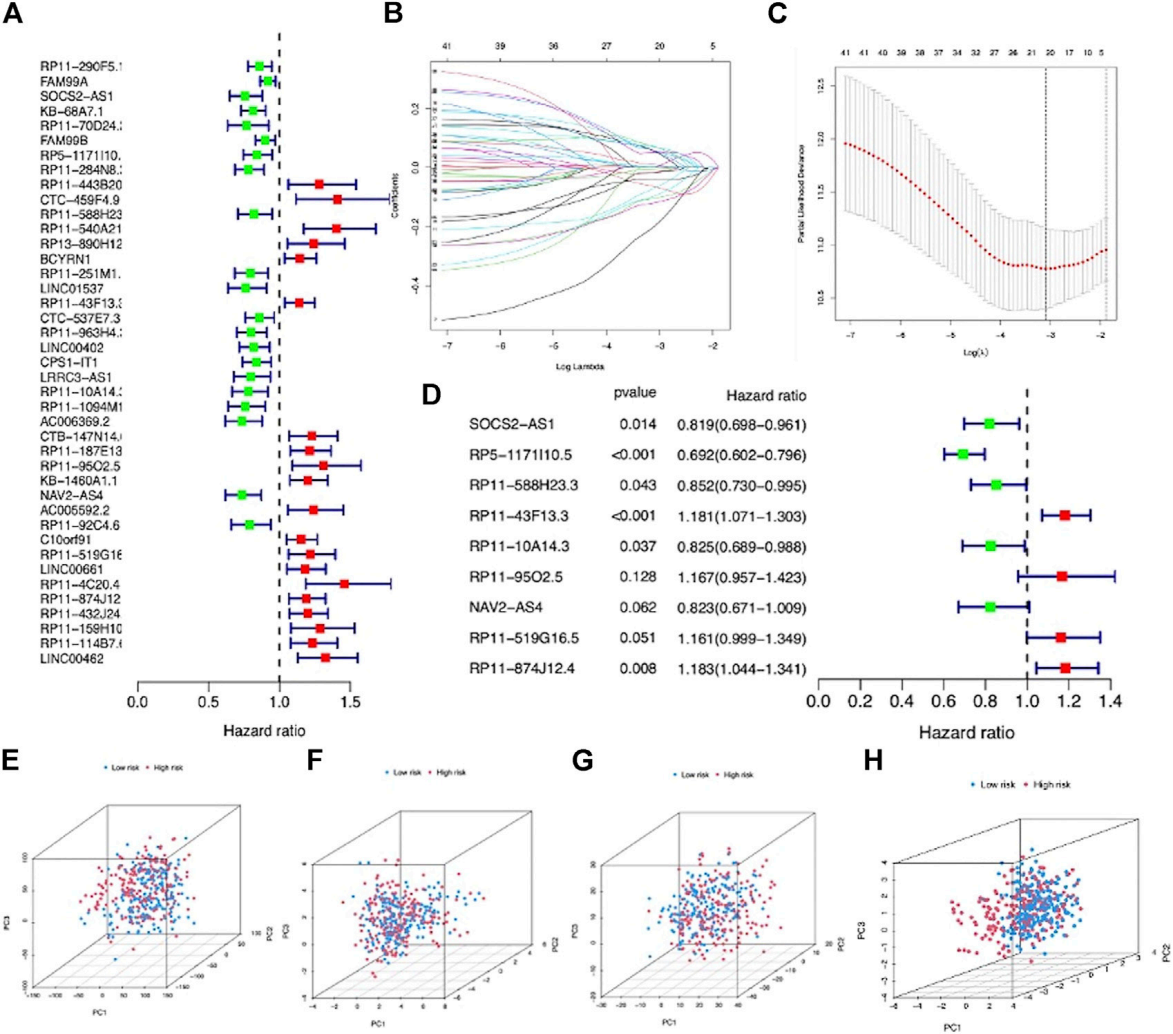
Construction of a m7G-related lncRNA risk model for LIHC patients. **(A)** Forest plot showed 41 prognostic lncRNAs screened *via* univariate regression analysis. **(B)** LASSO regression of 20 m7G-related lncRNAs. **(C)** Cross-validation in LASSO regression. **(D)** Forest plot displayed 9 m7G-related lncRNAs selected by multivariate regression analysis. **(E)** PCA based on entire LIHC gene expression profiles in the two groups. **(F)** PCA based on 22 m7G regulator gene expressions in the two groups. **(G)** PCA based on 992 m7G-related lncRNA expressions in the two groups. **(H)** PCA based on 9 prognostic m7G-related lncRNA expressions in the two groups.

The entire LIHC patients were separated into a training set (*n* = 219) and testing set (*n* = 146) in a random manner, and the clinical features are comparable between these two sets (*p* > 0.05) (Supplementary Table S1). First, 41 prognostic lncRNAs were selected from 992 m7G-related lncRNAs using univariate regression analysis in the training set, which revealed to be correlated with OS (*p* < 0.05), and half of them (20/41) were risk factors (hazard ratio, HR > 1) in LIHC (Figure 3A). Next, 20 m7G-related lncRNAs were further screened by LASSO regression analysis (Figures 3B,C), which can effectively reduce characteristics in high-dimensional data and optimize indicators predicting clinical outcome. Finally, a 9-m7G-related-lncRNA prognostic risk model was constructed by multivariate regression analysis (Figure 3D). The risk score was computed in the following manner: risk score = (−0.199 × SOCS2-AS1 expression) + (−0.368 × RP5-1171I10.5 expression) + (−0.160 × RP11-588H23.3 expression) + (0.167 × RP11-43F13.3 expression) + (−0.192 × RP11-10A14.3 expression) + (0.154202559 × RP11-95O2.5 expression) + (−0.194843224 × NAV2-AS4 expression) + (0.149322014 × RP11-519G16.5 expression) + (0.168349973 × RP11-874J12.4 expression). The correlation between m7G regulator genes and 9 m7G-related lncRNA expressions was also visualized in Supplementary Figure S1. Moreover, LIHC patients in the entire set were categorized into high- and low-risk groups based on the median risk score, and the grouping ability of this prognostic risk model was verified by PCA analysis. Results showed that the expression of the entire genes, 22 m7G regulator genes and 992 m7G-related lncRNAs, showed diffused distribution in both the risk groups (Figures 3E–G), whereas the expression of 9 m7G-related lncRNAs included in this prognostic risk model was well divided into two clusters with different risks (Figure 3H).

**FIGURE 4:**
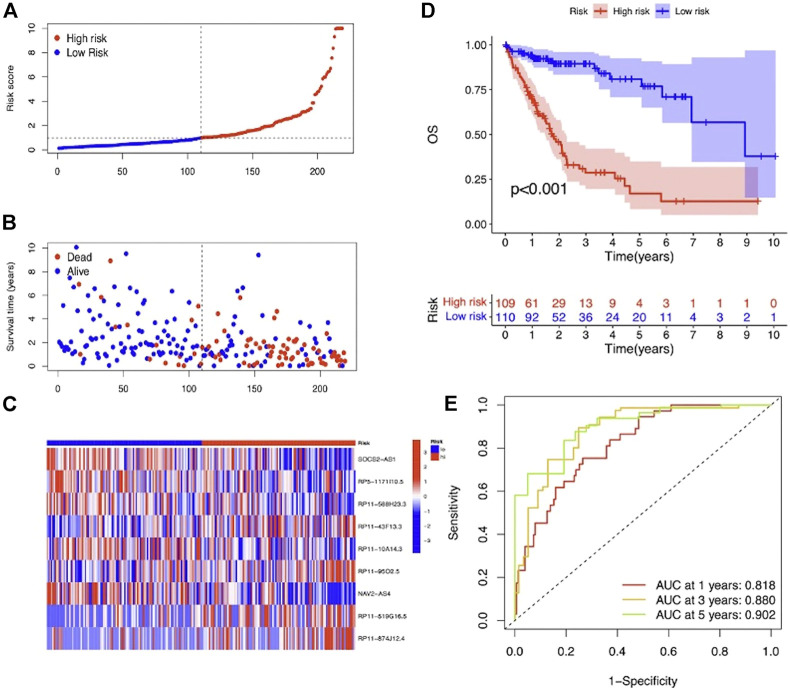
Prognostic value of the 9-m7G-related-lncRNA risk model between the two groups in the training set. **(A)** Distribution of the risk score (the x-axis represented the LIHC patients arranged according to the risk score, and the y-axis represented values of the risk score for each patient). **(B)** Scatter plot of survival status and risk score (the x-axis represented the LIHC patients arranged according to the risk score, and the y-axis represented the survival time of each patient). **(C)** Heatmap of the expression profile of the 9 m7G-related lncRNAs. **(D)** KM curves displayed the OS of LIHC patients between high- and low-risk groups. **(E)** ROC curves of the risk model of 1, 3, and 5 years for OS.

**FIGURE 5:**
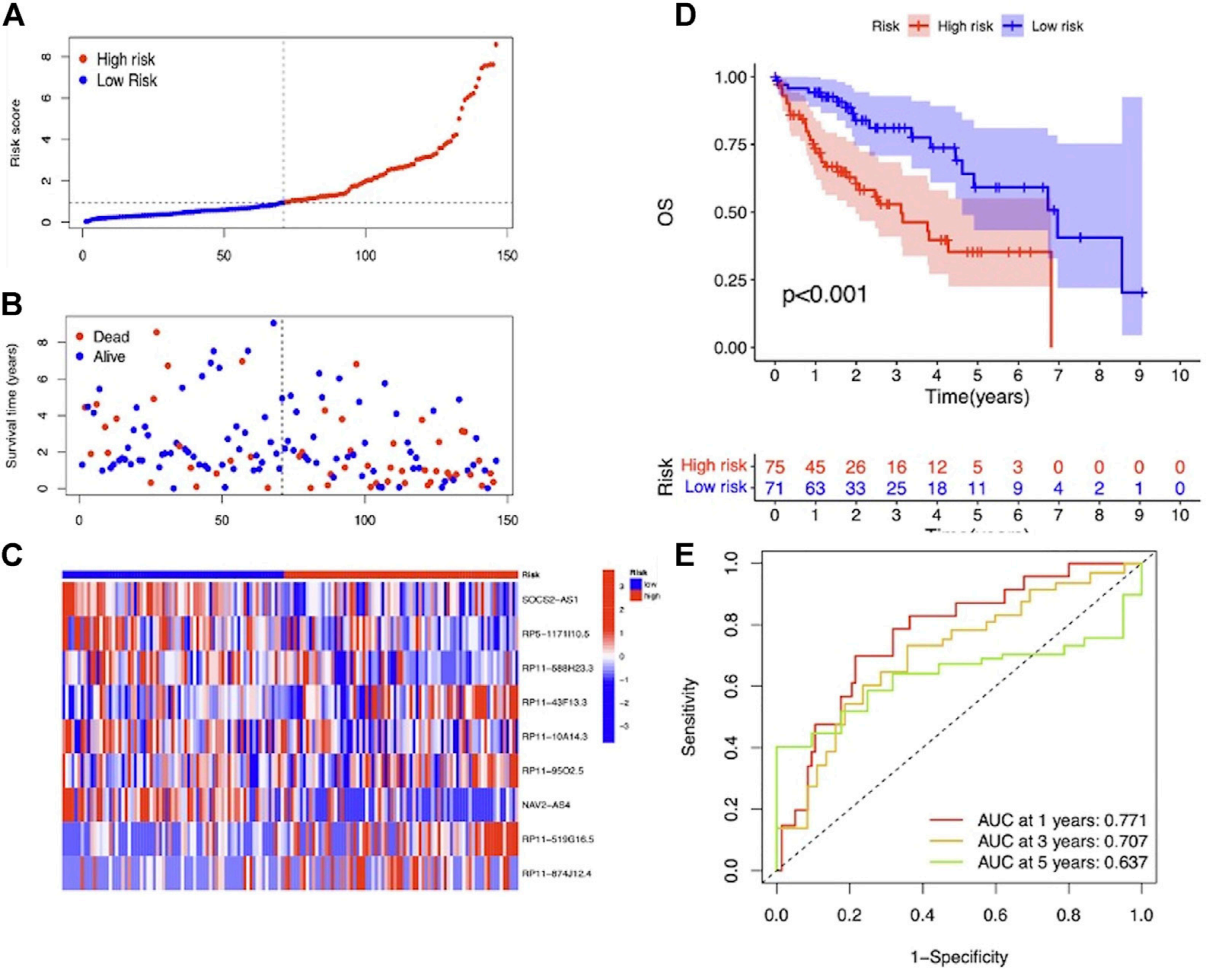
Prognostic value of the 9-m7G-related-lncRNA risk model between the two groups in the testing set. **(A)** Distribution of the risk score (the x-axis represented the LIHC patients arranged according to the risk score, and the y-axis represented values of the risk score for each patient). **(B)** Scatter plot of survival status and risk score (the x-axis represented the LIHC patients arranged according to the risk score, and the y-axis represented the survival time of each patient). **(C)** Heatmap of the expression profile of the 9 m7G-related lncRNAs. **(D)** KM curves displayed the OS of LIHC patients between high- and low-risk groups. **(E)** ROC curves of the risk model of 1, 3, and 5 years for OS.

To evaluate the prognostic efficacy of this model, we applied this risk score formula into different datasets. For the training set, we arranged the LIHC patients according to the risk score, and the heatmap showed that the expression pattern of the 9 m7G-related lncRNAs was different, and the scatter plot revealed that patients with a higher risk score featured worser living status (Figures 4A–C). The KM survival analysis indicated that high-risk patients had shorter OS than low-risk patients (*p* < 0.001) (Figure 4D). In addition, the predicting performance of the risk model was calculated with ROC curves, and AUROC of 1, 3, and 5 years for OS was 0.818, 0.880, and 0.902, respectively (Figure 4E). For the testing and entire set, the risk score distribution, heatmap of lncRNA expression, and scatter plot of survival status and risk score are depicted in Figures 5A–C and Supplementary Figures S2A–C, respectively. The survival analysis also showed that high-risk patients had shorter OS than low-risk patients in both testing and entire sets (Figure 5D; Supplementary Figure S1D). The AUROC of 1, 3, and 5 years for OS in the testing set was 0.771, 0.707, and 0.637 (Figure 5E) and in the entire set was 0.801, 0.811, and 0.777 (Supplementary Figure S2E). To further validate the capability of the prognostic risk model, we explored the difference in DSS, DFI, and PFI between the two groups and found that DSS, DFI, and PFI was also longer in low-risk patients (Supplementary Figures S3A–F). Taken together, the 9-m7G-related-lncRNA risk model possessed optimal prognostic efficacy in LIHC patients.

### Risk Score Based on the 9-m7G-Related-lncRNA Risk Model Was an Independent Prognostic Factor in LIHC Patients

**FIGURE 6:**
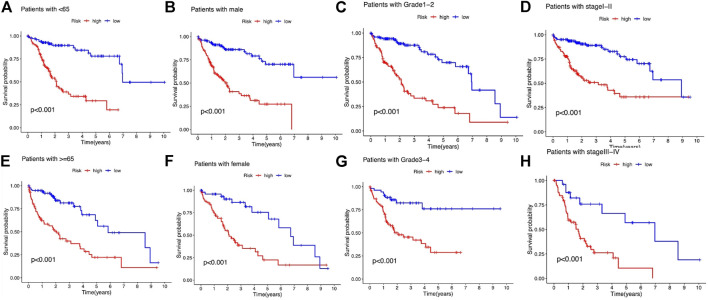
Kaplan–Meier survival analysis stratified by age, gender, tumor grade, and stage between the high- and low-risk groups in the entire set. **(A)** Patients with age <65. **(B)** Patients with male gender. **(C)** Patients with tumor grade 1-2. **(D)** Patients with tumor stage Ⅰ–Ⅱ. **(E)** Patients with age ≥65. **(F)** Patients with female gender. **(G)** Patients with tumor grade 3-4. **(H)** Patients with tumor stage Ⅲ–Ⅳ.

We subsequently stratified the clinicopathological features of LIHC patients according to the median value of the risk score and analyzed the difference in OS between high- and low-risk groups in the entire set. The survival analysis revealed that this risk model performed well in all the subgroups stratified by age (<65 years old and ≥65 years old), gender (male and female), tumor grade (grades 1–2 and grades 3–4), and stage (stages Ⅰ–Ⅱ and stages Ⅲ–Ⅳ), and high-risk patients had a shorter OS than the low-risk group (Figures 6A–H).

**FIGURE 7:**
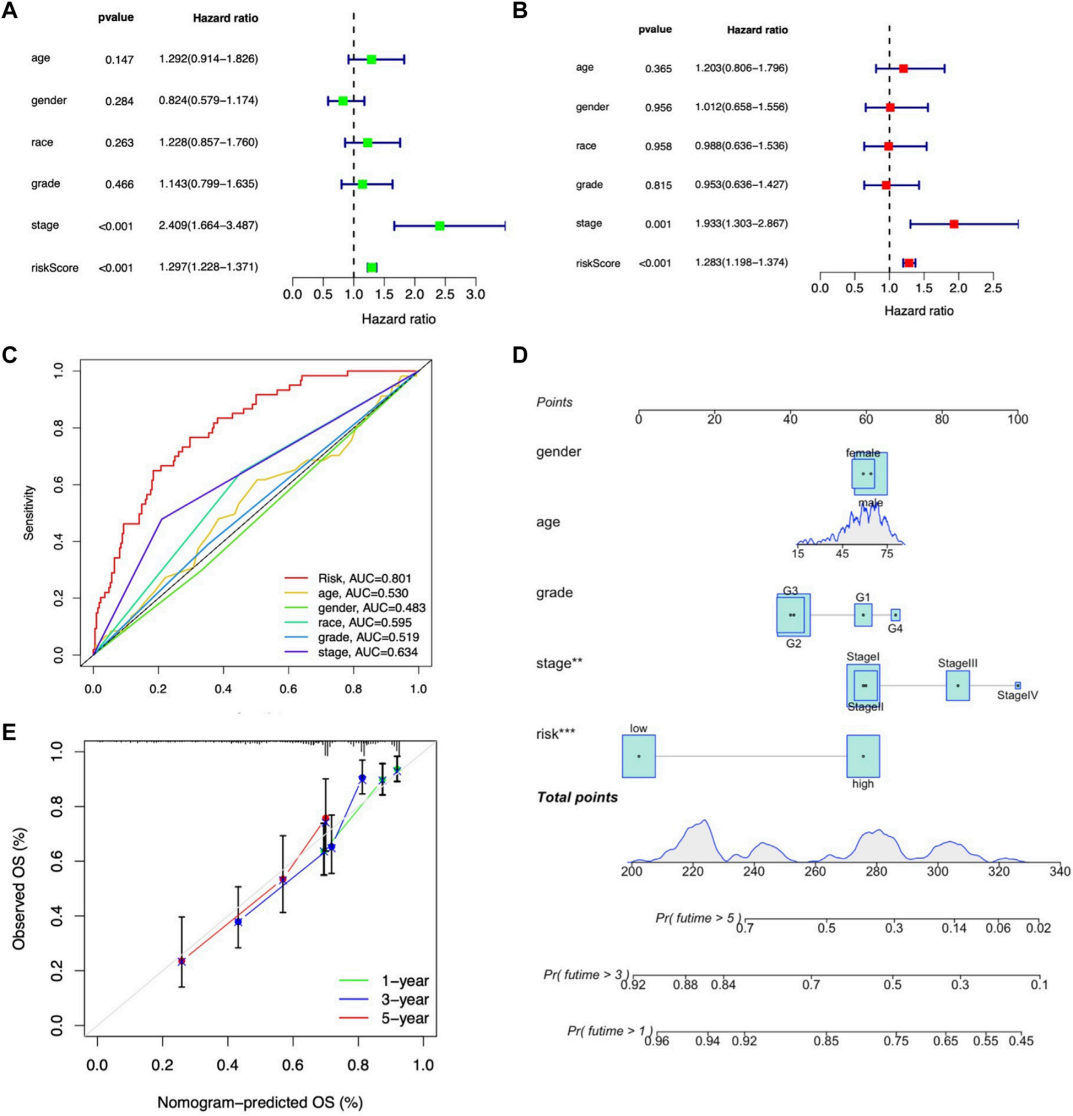
Assessment of the independent prognostic factors and construction of a prognostic nomogram in the entire LIHC set. **(A)** Univariate Cox regression analysis of the clinical characteristics and risk score with the OS. **(B)** Multivariate Cox regression analysis of the clinical characteristics and risk score with the OS. **(D)** Nomogram predicting the probability of 1-, 2-, and 3-year OS. **(C)** ROC curves of the clinical characteristics and risk score. **(E)** Calibration plot of the nomogram predicting the probability of the 1-, 2-, and 3-year OS.

To further validate the independent prognostic factors in LIHC patients, univariate and multivariate Cox regression analyses were performed. Univariate Cox regression analysis indicated that both stage (HR: 2.409%, 95% CI: 1.664–3.487, *p* < 0.001) and m7G-related lncRNA risk score (HR: 1.297%, 95% CI: 1.228–1.371, *p* < 0.001) were significantly related with OS (Figure 7A). Multivariate analysis also revealed that stage (HR: 1.933%, 95% CI: 1.303–1.867, *p* < 0.001) and m7G-related lncRNA risk score (HR: 1.283%, 95% CI: 1.198–1.374, *p* < 0.001) were independent prognostic factors in LIHC patients (Figure 7B). The AUROC was calculated to better explicit the efficacy of m7G-related lncRNA risk score as well as other clinicopathological features in predicting the OS in LIHC patients, and results demonstrated that the risk score had higher AUROC than other clinicopathological features (Figure 7C).

Moreover, we constructed the nomogram consisting of clinicopathological features (including age, gender, tumor grade, and stage) along with the m7G-related lncRNA risk score to predict the survival status in entire LIHC patients. Both m7G-related lncRNA risk score and tumor stage showed better predictive ability than other clinicopathological features in the nomogram (Figure 7D). The calibration curve also proved acceptable consistency between the actual and predicted survival within 1-, 3-, and 5-year OS (Figure 7E), demonstrating that the 9-m7G-related-lncRNA risk model was reliable and could work well in predicting prognosis in LIHC patients.

### Analysis of Tumor Mutation Burden, Immune Landscape, and Immunotherapy Response Targeting the m7G-Related lncRNA Risk Model

**FIGURE 8:**
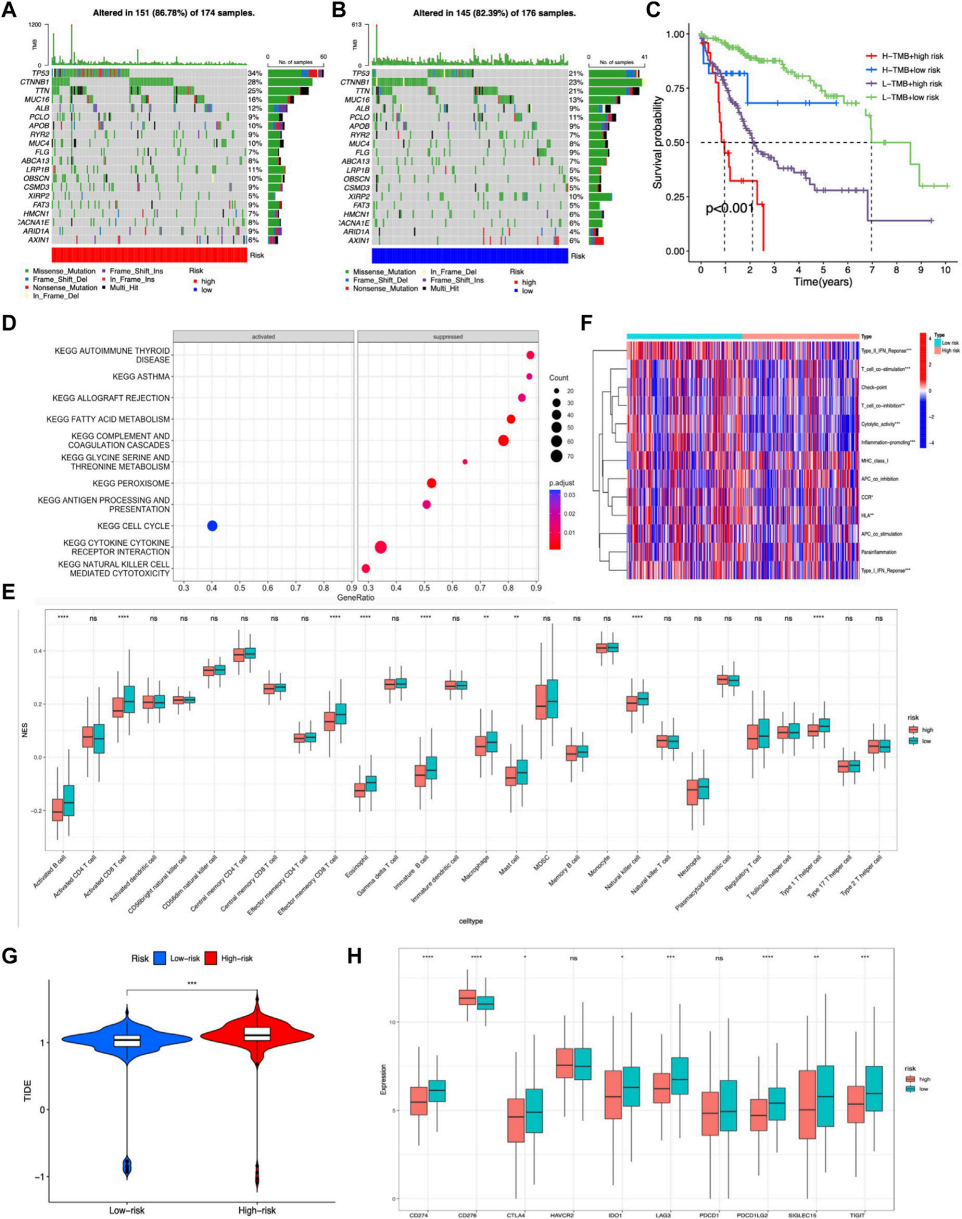
Evaluation of the tumor immune landscape and immunotherapy response based on the m7G-related lncRNA model in the entire LIHC set. **(A,B)** Waterfall plot displayed top 20 mutation genes’ information in the two risk groups. **(C)** KM survival analysis of OS stratified by tumor mutation burden and the m7G-related lncRNA model. **(G)** TIDE prediction difference between two risk score subgroups. **(D)** Significant KEGG pathways enriched in high-risk patients. **(E)** Difference in tumor infiltration immune cells based on ssGSEA scores between two risk groups. **(F)** Difference in immune-related functions based on ssGSEA scores between two risk score subgroups. **(H)** Expression of immune checkpoint blockade-related genes between the two risk groups.

TMB was calculated by using the R package “maftools” in the two risk groups, and results showed that gene mutation incidence was elevated in the high-risk group, especially TP53 gene (34% vs. 21%) (Figures 8A,B). Survival analysis demonstrated that the OS outcome was shorter in high-risk patients regardless of the TMB risk. Furthermore, patients with high TMB in the low-risk group survived longer than patients with low TMB in high-risk group, indicating that the m7G-related lncRNA risk model had better prognostic significance than TMB status (Figure 8C).

The tumor immune landscape, including pathway enrichment, immune cell infiltration, and immune-related functions, was analyzed based on the m7G-related lncRNA risk model in LIHC patients with different risks. First, KEGG pathway analysis was performed to explore the underlying molecular mechanisms occurred in different risk groups, and results illustrated that many immune-related pathways were markedly suppressed in the high-risk group, such as antigen processing and presentation, cytokine–receptor interaction, and natural killer cell-mediated cytotoxicity, while only cell cycle pathway was significantly activated (Figure 8D). Next, the tumor infiltration immune cells and immune-related functions were assessed based on ssGSEA scores between the two risk groups, and results showed that activated B cells, immature B cells, memory B cells, activated CD8^+^ T cells, effector memory CD8^+^ T cells, natural killer T cells, Th1 cells, eosinophil, macrophage, and mast cells exhibited a lower expression in the high-risk group (Figure 8E). Additionally, the heatmap showed many immune-related functions, such as type2 IFN response, T-cell co-stimulation, T-cell co-inhibition, cytolytic activity, inflammation promoting, CCR, HLA, and type1 IFN response, were also downregulated in the high-risk group (Figure 8F).

As for immunotherapy response, previous studies had demonstrated that a higher TIDE score was associated with worse immunotherapy response and poor prognosis (Jiang et al., 2018). Hence, we calculated the TIDE score to predict the response to immunotherapy in LIHC patients and disclosed that the high-risk group had a lower response rate to immunotherapy than the low-risk group, which was in accordance with previous tumor immune microenvironment analysis (Figure 8G). Moreover, immune checkpoint molecules are vital targets of immune checkpoint inhibitors (ICIs), and we discovered that high-risk group patients exhibited lower expression of CD274, PDCD1LG2, LAG3, SIGLEC15, TIGIT, IDO1, and CTLA4 than low-risk group patients, except for CD276 (Figure 8H).

Furthermore, novel candidate compounds based on the m7G-related lncRNA risk model were identified by calculating the IC_50_ for each LIHC patient using the GDSC database. In total, 47 of 138 compounds were found to exhibit significant differences in the estimated IC_50_ between the two risk groups (*p* < 0.05), suggesting that the m7G-related lncRNA risk model had the potential of predicting the chemotherapy sensitivity. Top 20 novel compounds are listed in Supplementary Figures S4A–T and could be used for further analysis in LIHC patients.

## Discussion

LIHC is a common malignancy with high morbidity and mortality, which seriously threatens the health of people all over the world. Clinically, for the reasons such as lacking effective approaches for early diagnosis, most patients have progressed to the advanced stage and then lost the opportunity of early eradication treatment. Hence, accurate biomarkers are needed to improve the diagnostic and treatment efficacy of LIHC patients.

LncRNAs as novel regulatory molecules play fundamental roles in the progression of LIHC (Ghafouri-Fard et al., 2021). Studies have shown that lncRNAs can modulate the proliferation, migration, invasion, angiogenesis, and drug resistance of tumor cells by forming a complex regulatory network with mRNA and miRNA (Huang Z. et al., 2020). Meanwhile, lncRNAs can also be used as indicators for early diagnosis and efficacy prediction of treatment strategies, such as surgery, radiotherapy, chemotherapy, and immunotherapy, thus becoming important molecular types for the development of diagnostic biomarkers as well as molecular therapeutic targets of LIHC (Yuan et al., 2021). In recent years, the methylation modification of lncRNA, such as m6A and m5C, has been revealed to be involved in tumor progression including LIHC, and RNA methylation-related lncRNAs have demonstrated to be novel potential biomarkers for tumor diagnosis and prognosis as well as immunotherapy response (Jin et al., 2021; Li et al., 2021; Zhu et al., 2021).

As a widespread RNA epigenetic modification in eukaryotes, m7G has recently been found to modify tRNA and promote tumor progression by regulating translation efficiency (Katsara and Schneider, 2021). For LIHC, METTL1, the key component of the m7G methyltransferase complex, was disclosed to be upregulated in LIHC and can promote tumor progression *via* m7G tRNA modification-dependent translation control (Chen et al., 2021). However, whether m7G modifications are involved in tumorigenesis and development by regulating lncRNAs remain unknown. Given the potential of m6A- and m5C-associated lncRNAs in predicting tumor prognosis and immunotherapy response, we speculate that m7G-related lncRNAs are highly likely to possess the same ability. In the present study, we constructed and validated the prognostic risk model based on m7G-related lncRNAs and explored the roles of the model in evaluating tumor mutation load, immune cell infiltration, immunotherapy response, and drug sensitivity.

To obtain m7G-related lncRNAs, we first locked 22 m7G regulatory genes through literature retrieval, including 2 “writers” (METTL1 and WDR4), 8 “erasers” (DCP2, DCPS, NUDT2, NUDT3, NUDT12, NUDT15, NUDT16, and NUDT17), and 12 “readers” (AGO2, CYFIP1, EIF4E, EIF4E2, EIF4E3, GEMIN5, LARP1, NCBP1, NCBP2, EIF3D, EIF4A1, and EIF4G3). Currently, apart from METTL1 and WDR4 (Chen et al., 2021; Xia et al., 2021), it is still unclear whether other genes can participate in LIHC progression *via* m7G-dependent manner. Here, we found that the majority of m7G regulators (16/22) were abnormally expressed in LIHC, indicating m7G modification may exert crucial functions in LIHC. Subsequently, a total of 992 lncRNAs that were co-expressed with m7G regulators were screened through Pearson correlation analysis.

Combined with clinicopathological data, an LIHC prognostic risk model comprising 9 m7G-related lncRNAs was finally achieved by univariate, LASSO, and multivariate Cox regression analyses successively. Five lncRNAs (SOCS2-AS1, RP5-1171I10.5, RP11-588H23.3, RP11-10A14.3, and NAV2-AS4) were identified to be a protective factor for LIHC prognosis, and other four lncRNAs (RP11-43F13.3, RP11-95O2.5, RP11-519G16.5, and RP11-874J12.4) were risk factors affecting the prognosis of LIHC. SOCS2-AS1 has recently been validated as a tumor suppressor in colorectal and endometrial cancers, and its low expression in tumors was positively related to poor prognosis of patients (Zheng et al., 2020; Jian et al., 2021). RP11-874J12.4 was recently identified as an oncogenic lncRNA that could facilitate oral squamous cell carcinoma tumorigenesis and gastric cancer chemoresistance (Liu et al., 2020; Liu et al., 2021). Subsequently, LIHC patients were separated into high- and low-risk groups based on the median risk score, and its prognostic efficacy was evaluated through KM and ROC analysis, and we found that the model showed good prognostic performance in all the datasets. Moreover, univariate and multivariate Cox regression analyses disclosed that the risk score based on the m7G-related lncRNA risk model was an independent prognostic factor for LIHC, and the m7G-related lncRNA risk score exhibited better OS prediction performance than other clinicopathological features, including age, gender, race, and tumor grade as well as stage.

Tumor mutation burden (TMB) is associated with genomic instability and immunogenicity, involving base substitutions, gene insertion and deletion, and other mutations and has the potential in predicting prognosis and immunotherapy efficacy (Huang et al., 2021). In this study, we investigated the TMB in different risk groups and found that TMB was elevated in the high-risk group, and LIHC patients with high TMB had worse OS in both the risk groups. Several studies also revealed the negative roles of TMB in the prognosis of LIHC (Cai et al., 2020; Zhou et al., 2021).

Tumor immune microenvironment (TIME) influences tumor progression and immunotherapy response. Although immunotherapy (such as immune checkpoint inhibitor drugs) has shown potential therapeutic effects for advanced LIHC, and the effectiveness of immunotherapy is greatly affected by its immunosuppressive TIME (Zhou et al., 2022). Currently, changing the TIME has become a new strategy to promote immune control and immunotherapy efficacy of tumors. In the present study, the relationship between m7G-related lncRNAs and TIME was evaluated by exploring KEGG pathway enrichment, immune cell infiltration, and immune-related functions. We found that high-risk LIHC patients were more inclined to an immunosuppressive state because immune-related pathways (such as natural killer cell-mediated cytotoxicity, antigen processing, and presentation), immune-related function (such as type1/2 IFN response, T-cell co-stimulation, and cytolytic activity), and anti-tumor immune cells (such as activated B cells, CD8^+^ T cells, and natural killer T cells) were all inactivated in high-risk patients, suggesting that m7G-related lncRNAs may be related to the immunosuppressive tumor microenvironment in LIHC. Furthermore, the association between m7G-related lncRNAs and immunotherapy response was also investigated by TIDE scoring, and we unveiled that high-risk LIHC patients showed a lower response rate to immunotherapy, indicating that m7G-related lncRNAs have great potential in predicting immunotherapy response. Additionally, the correlation between the m7G-related lncRNA risk model and the sensitivity of 138 anti-cancer drugs was evaluated using GDSC database, and 47 compounds were identified to exhibit the difference in chemotherapy sensitivity between the two groups, such as cisplatin, cytarabine, tipifarnib, and temsirolimus, suggesting that m7G-related lncRNA may also be used as a biomarker to predict the chemotherapy sensitivity and provide medication guidance for the personalized chemotherapy of LIHC patients.

## Conclusion

We constructed a 9-m7G-related-lncRNA risk model based on the LIHC patients’ clinical and transcriptomic data from TCGA database. The risk model was validated to exhibit good prognostic performance and was found to be an independent prognostic factor for LIHC. Furthermore, the roles of the risk model in tumor mutation burden, immune microenvironment, and immunotherapy response, as well as drug sensitivity were also evaluated. In conclusion, the m7G-related-lncRNA risk model might display potential value in predicting prognosis, immunotherapy response, and drug sensitivity in LIHC patients.

## Data Availability

The original contributions presented in the study are included in the article/Supplementary Material; further inquiries can be directed to the corresponding author.

## References

[B1] Bradrick S. S. Gromeier M. (2009). Identification of Gemin5 as a Novel 7-Methylguanosine Cap-Binding Protein. PLoS One 4 (9), e7030. 10.1371/journal.pone.0007030 19750007 PMC2736588

[B2] Cai H. Zhang Y. Zhang H. Cui C. Li C. Lu S. (2020). Prognostic Role of Tumor Mutation Burden in Hepatocellular Carcinoma after Radical Hepatectomy. J. Surg. Oncol. 121 (6), 1007–1014. 10.1002/jso.25859 31995247

[B3] Chen Z. Zhu W. Zhu S. Sun K. Liao J. Liu H. (2021). METTL1 Promotes Hepatocarcinogenesis via m(7) G tRNA Modification-Dependent Translation Control. Clin. Transl. Med. 11 (12), e661. 10.1002/ctm2.661 34898034 PMC8666584

[B4] Chu J. Zhang W. Cencic R. O’Connor P. B. F. Robert F. Devine W. G. (2020). Rocaglates Induce Gain-Of-Function Alterations to eIF4A and eIF4F. Cell Rep. 30 (8), 2481–2488. 10.1016/j.celrep.2020.02.002 32101697 PMC7077502

[B5] Dou Y. Kalmykova S. Pashkova M. Oghbaie M. Jiang H. Molloy K. R. (2020). Affinity Proteomic Dissection of the Human Nuclear Cap-Binding Complex Interactome. Nucleic Acids Res. 48 (18), 10456–10469. 10.1093/nar/gkaa743 32960270 PMC7544204

[B6] Gao N. Li Y. Li J. Gao Z. Yang Z. Li Y. (2020). Long Non-Coding RNAs: The Regulatory Mechanisms, Research Strategies, and Future Directions in Cancers. Front. Oncol. 10, 598817. 10.3389/fonc.2020.598817 33392092 PMC7775490

[B7] Ghafouri-Fard S. Gholipour M. Hussen B. M. Taheri M. (2021). The Impact of Long Non-Coding RNAs in the Pathogenesis of Hepatocellular Carcinoma. Front. Oncol. 11, 649107. 10.3389/fonc.2021.649107 33968749 PMC8097102

[B8] Haghighat A. Sonenberg N. (1997). eIF4G Dramatically Enhances the Binding of eIF4E to the mRNA 5'-cap Structure. J. Biol. Chem. 272 (35), 21677–21680. 10.1074/jbc.272.35.21677 9268293

[B9] Huang A. Yang X.-R. Chung W.-Y. Dennison A. R. Zhou J. (2020). Targeted Therapy for Hepatocellular Carcinoma. Sig. Transduct. Target. Ther. 5 (1), 146. 10.1038/s41392-020-00264-x PMC7419547 32782275

[B10] Huang T. Chen X. Zhang H. Liang Y. Li L. Wei H. (2021). Prognostic Role of Tumor Mutational Burden in Cancer Patients Treated with Immune Checkpoint Inhibitors: A Systematic Review and Meta-Analysis. Front. Oncol. 11, 706652. 10.3389/fonc.2021.706652 34395281 PMC8358612

[B11] Huang Z. Zhou J.-K. Peng Y. He W. Huang C. (2020). The Role of Long Noncoding RNAs in Hepatocellular Carcinoma. Mol. Cancer 19 (1), 77. 10.1186/s12943-020-01188-4 32295598 PMC7161154

[B12] Jian F. Che X. Zhang J. Liu C. Liu G. Tang Y. (2021). The Long-Noncoding RNA SOCS2-AS1 Suppresses Endometrial Cancer Progression by Regulating AURKA Degradation. Cell Death Dis. 12 (4), 351. 10.1038/s41419-021-03595-x 33824269 PMC8024384

[B13] Jiang P. Gu S. Pan D. Fu J. Sahu A. Hu X. (2018). Signatures of T Cell Dysfunction and Exclusion Predict Cancer Immunotherapy Response. Nat. Med. 24 (10), 1550–1558. 10.1038/s41591-018-0136-1 30127393 PMC6487502

[B14] Jin C. Li R. Deng T. Li J. Yang Y. Li H. (2021). Identification and Validation of a Prognostic Prediction Model of m6A Regulator-Related LncRNAs in Hepatocellular Carcinoma. Front. Mol. Biosci. 8, 784553. 10.3389/fmolb.2021.784553 34988119 PMC8721125

[B15] Kamarajah S. K. Bundred J. R. Littler P. Reeves H. Manas D. M. White S. A. (2021). Treatment Strategies for Early Stage Hepatocellular Carcinoma: A Systematic Review and Network Meta-Analysis of Randomised Clinical Trials. HPB 23 (4), 495–505. 10.1016/j.hpb.2020.10.031 33309569

[B16] Katsara O. Schneider R. J. (2021). m(7)G tRNA Modification Reveals New Secrets in the Translational Regulation of Cancer Development. Mol. Cell 81 (16), 3243–3245. 10.1016/j.molcel.2021.07.030 34416137 PMC10883294

[B17] Kiriakidou M. Tan G. S. Lamprinaki S. De Planell-Saguer M. Nelson P. T. Mourelatos Z. (2007). An mRNA m7G Cap Binding-Like Motif within Human Ago2 Represses Translation. Cell 129 (6), 1141–1151. 10.1016/j.cell.2007.05.016 17524464

[B18] Lee A. S. Y. Kranzusch P. J. Doudna J. A. Cate J. H. D. (2016). eIF3d is an mRNA Cap-Binding Protein that is Required for Specialized Translation Initiation. Nature 536 (7614), 96–99. 10.1038/nature18954 27462815 PMC5003174

[B19] Li L. Xie R. Lu G. (2021). Identification of m6A Methyltransferase-Related lncRNA Signature for Predicting Immunotherapy and Prognosis in Patients with Hepatocellular Carcinoma. Biosci. Rep. 41 (6), BSR20210760. 10.1042/BSR20210760 34027555 PMC8188173

[B20] Liu G. M. Lu T. C. Sun M. L. Ji X. Zhao Y. A. Jia W. Y. (2020). RP11‐874J12.4 Promotes Oral Squamous Cell Carcinoma Tumorigenesis via the miR‐19a‐5p/EBF1 Axis. J. Oral Pathol. Med. 49 (7), 645–654. 10.1111/jop.13000 32004389

[B21] Liu Y. Cao J. Pu Y. S. Ma Y. Wu M. Wang J. H. (2021). RP11-874J12.4, a Novel lncRNA, Confers Chemoresistance in Human Gastric Cancer Cells by Sponging miR-3972 and Upregulating SSR2 Expression. Am. J. Transl. Res. 13 (6), 5892–5910. 34306333 PMC8290636

[B22] Llovet J. M. Castet F. Heikenwalder M. Maini M. K. Mazzaferro V. Pinato D. J. (2022). Immunotherapies for Hepatocellular Carcinoma. Nat. Rev. Clin. Oncol. 19 (3), 151–172. 10.1038/s41571-021-00573-2 34764464

[B23] Llovet J. M. Kelley R. K. Villanueva A. Singal A. G. Pikarsky E. Roayaie S. (2021). Hepatocellular Carcinoma. Nat. Rev. Dis. Prim. 7 (1), 6. 10.1038/s41572-020-00240-3 33479224 PMC13537433

[B24] Luo D. Deng B. Weng M. Luo Z. Nie X. (2018). A Prognostic 4-lncRNA Expression Signature for Lung Squamous Cell Carcinoma. Artif. Cells Nanomed. Biotechnol. 46 (6), 1207–1214. 10.1080/21691401.2017.1366334 28835135

[B25] Ma J. Han H. Huang Y. Yang C. Zheng S. Cai T. (2021). METTL1/WDR4-Mediated m(7)G tRNA Modifications and m(7)G Codon Usage Promote mRNA Translation and Lung Cancer Progression. Mol. Ther. 29 (12), 3422–3435. 10.1016/j.ymthe.2021.08.005 34371184 PMC8636169

[B26] Malbec L. Zhang T. Chen Y.-S. Zhang Y. Sun B.-F. Shi B.-Y. (2019). Dynamic Methylome of Internal mRNA N(7)-Methylguanosine and its Regulatory Role in Translation. Cell Res. 29 (11), 927–941. 10.1038/s41422-019-0230-z 31520064 PMC6889513

[B27] Napoli I. Mercaldo V. Boyl P. P. Eleuteri B. Zalfa F. De Rubeis S. (2008). The Fragile X Syndrome Protein Represses Activity-Dependent Translation through CYFIP1, a New 4E-BP. Cell 134 (6), 1042–1054. 10.1016/j.cell.2008.07.031 18805096

[B28] Orellana E. A. Liu Q. Yankova E. Pirouz M. De Braekeleer E. Zhang W. (2021). METTL1-Mediated m(7)G Modification of Arg-TCT tRNA Drives Oncogenic Transformation. Mol. Cell 81 (16), 3323–3338. e3314. 10.1016/j.molcel.2021.06.031 34352207 PMC8380730

[B29] Osborne M. J. Volpon L. Kornblatt J. A. Culjkovic-Kraljacic B. Baguet A. Borden K. L. B. (2013). eIF4E3 Acts as a Tumor Suppressor by Utilizing an Atypical Mode of Methyl-7-Guanosine Cap Recognition. Proc. Natl. Acad. Sci. U. S. A. 110 (10), 3877–3882. 10.1073/pnas.1216862110 23431134 PMC3593863

[B30] Oura K. Morishita A. Tani J. Masaki T. (2021). Tumor Immune Microenvironment and Immunosuppressive Therapy in Hepatocellular Carcinoma: A Review. Int. J. Mol. Sci. 22 (11), 5801. 10.3390/ijms22115801 34071550 PMC8198390

[B31] Pandolfini L. Barbieri I. Bannister A. J. Hendrick A. Andrews B. Webster N. (2019). METTL1 Promotes Let-7 MicroRNA Processing via m7G Methylation. Mol. Cell 74 (6), 1278–1290. 10.1016/j.molcel.2019.03.040 31031083 PMC6591002

[B32] Philippe L. Vasseur J.-J. Debart F. Thoreen C. C. (2018). La-Related Protein 1 (LARP1) Repression of TOP mRNA Translation is Mediated through its Cap-Binding Domain and Controlled by an Adjacent Regulatory Region. Nucleic Acids Res. 46 (3), 1457–1469. 10.1093/nar/gkx1237 29244122 PMC5814973

[B33] Rosettani P. Knapp S. Vismara M.-G. Rusconi L. Cameron A. D. (2007). Structures of the Human eIF4E Homologous Protein, h4EHP, in its m7GTP-Bound and Unliganded Forms. J. Mol. Biol. 368 (3), 691–705. 10.1016/j.jmb.2007.02.019 17368478

[B34] Song M.-G. Bail S. Kiledjian M. (2013). Multiple Nudix Family Proteins Possess mRNA Decapping Activity. RNA 19 (3), 390–399. 10.1261/rna.037309.112 23353937 PMC3677249

[B35] Statello L. Guo C.-J. Chen L.-L. Huarte M. (2021). Gene Regulation by Long Non-Coding RNAs and its Biological Functions. Nat. Rev. Mol. Cell Biol. 22 (2), 96–118. 10.1038/s41580-020-00315-9 33353982 PMC7754182

[B36] Tian Q.-H. Zhang M.-F. Zeng J.-S. Luo R.-G. Wen Y. Chen J. (2019). METTL1 Overexpression is Correlated with Poor Prognosis and Promotes Hepatocellular Carcinoma via PTEN. J. Mol. Med. 97 (11), 1535–1545. 10.1007/s00109-019-01830-9 31463732

[B37] Wang H. Chen R. B. Zhang S. N. Zhang R. F. (2022). N7-Methylguanosine Modification of lncRNAs in a Rat Model of Hypoxic Pulmonary Hypertension: A Comprehensive Analysis. BMC Genomics 23 (1), 33. 10.1186/s12864-021-08188-8 34996349 PMC8740322

[B38] Wang T. Kong S. Tao M. Ju S. (2020). The Potential Role of RNA N6-Methyladenosine in Cancer Progression. Mol. Cancer 19 (1), 88. 10.1186/s12943-020-01204-7 32398132 PMC7216508

[B39] Wong J. S. L. Kwok G. G. W. Tang V. Li B. C. W. Leung R. Chiu J. (2021). Ipilimumab and Nivolumab/Pembrolizumab in Advanced Hepatocellular Carcinoma Refractory to Prior Immune Checkpoint Inhibitors. J. Immunother. Cancer 9 (2), e001945. 10.1136/jitc-2020-001945 33563773 PMC7875295

[B40] Wulf M. G. Buswell J. Chan S.-H. Dai N. Marks K. Martin E. R. (2019). The Yeast Scavenger Decapping Enzyme DcpS and its Application for *In Vitro* RNA Recapping. Sci. Rep. 9 (1), 8594. 10.1038/s41598-019-45083-5 31197197 PMC6565619

[B41] Wurm J. P. Sprangers R. (2019). Dcp2: An mRNA Decapping Enzyme that Adopts Many Different Shapes and Forms. Curr. Opin. Struct. Biol. 59, 115–123. 10.1016/j.sbi.2019.07.009 31473440 PMC6900585

[B42] Xia P. Zhang H. Xu K. Jiang X. Gao M. Wang G. (2021). MYC-Targeted WDR4 Promotes Proliferation, Metastasis, and Sorafenib Resistance by Inducing CCNB1 Translation in Hepatocellular Carcinoma. Cell Death Dis. 12 (7), 691. 10.1038/s41419-021-03973-5 34244479 PMC8270967

[B43] Yuan D. Chen Y. Li X. Li J. Zhao Y. Shen J. (2021). Long Non-Coding RNAs: Potential Biomarkers and Targets for Hepatocellular Carcinoma Therapy and Diagnosis. Int. J. Biol. Sci. 17 (1), 220–235. 10.7150/ijbs.50730 33390845 PMC7757045

[B44] Zhang Q. Liu F. Chen W. Miao H. Liang H. Liao Z. (2021). The Role of RNA m(5)C Modification in Cancer Metastasis. Int. J. Biol. Sci. 17 (13), 3369–3380. 10.7150/ijbs.61439 34512153 PMC8416729

[B45] Zhao L. Wang J. Li Y. Song T. Wu Y. Fang S. (2021). NONCODEV6: an Updated Database Dedicated to Long Non-Coding RNA Annotation in Both Animals and Plants. Nucleic Acids Res. 49 (D1), D165–D171. 10.1093/nar/gkaa1046 33196801 PMC7779048

[B46] Zheng Z. Li X. You H. Zheng X. Ruan X. (2020). LncRNA SOCS2-AS1 Inhibits Progression and Metastasis of Colorectal Cancer through Stabilizing SOCS2 and Sponging miR-1264. Aging 12 (11), 10517–10526. 10.18632/aging.103276 32437330 PMC7346041

[B47] Zhou G. Boor P. P. C. Bruno M. J. Sprengers D. Kwekkeboom J. (2022). Immune Suppressive Checkpoint Interactions in the Tumour Microenvironment of Primary Liver Cancers. Br. J. Cancer 126 (1), 10–23. 10.1038/s41416-021-01453-3 34400801 PMC8727557

[B48] Zhou W. Fang D. He Y. Wei J. (2021). Correlation Analysis of Tumor Mutation Burden of Hepatocellular Carcinoma Based on Data Mining. J. Gastrointest. Oncol. 12 (3), 1117–1131. 10.21037/jgo-21-259 34295561 PMC8261312

[B49] Zhu X.-L. Li Q. Shen J. Shan L. Zuo E.-D. Cheng X. (2021). Use of 6 m6A-Relevant lncRNA Genes as Prognostic Markers of Primary Liver Hepatocellular Carcinoma Based on TCGA Database. Transl. Cancer Res. TCR 10 (12), 5337–5351. 10.21037/tcr-21-2440 35116381 PMC8797289

