# Supplementary figures and images for: Prognostic Signature and Tumor Immune Landscape of N7-Methylguanosine-Related lncRNAs in Hepatocellular Carcinoma
Source: Front Genet. 2022 Jul 22;13:906496. doi: 10.3389/fgene.2022.906496 (PMC9354608; doi:10.3389/fgene.2022.906496)

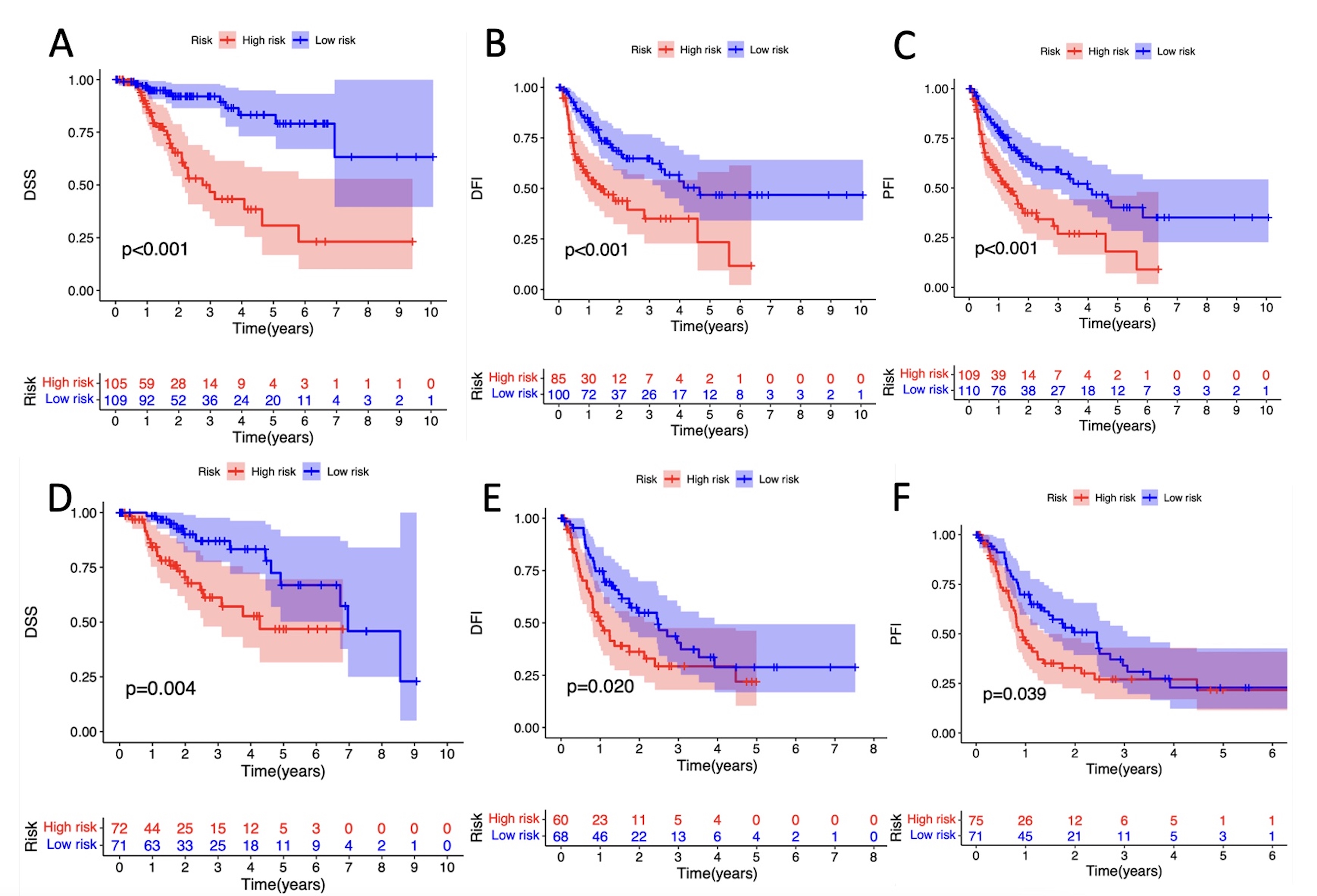

Supplement: Supplementary file 1 [file Image3.JPEG]

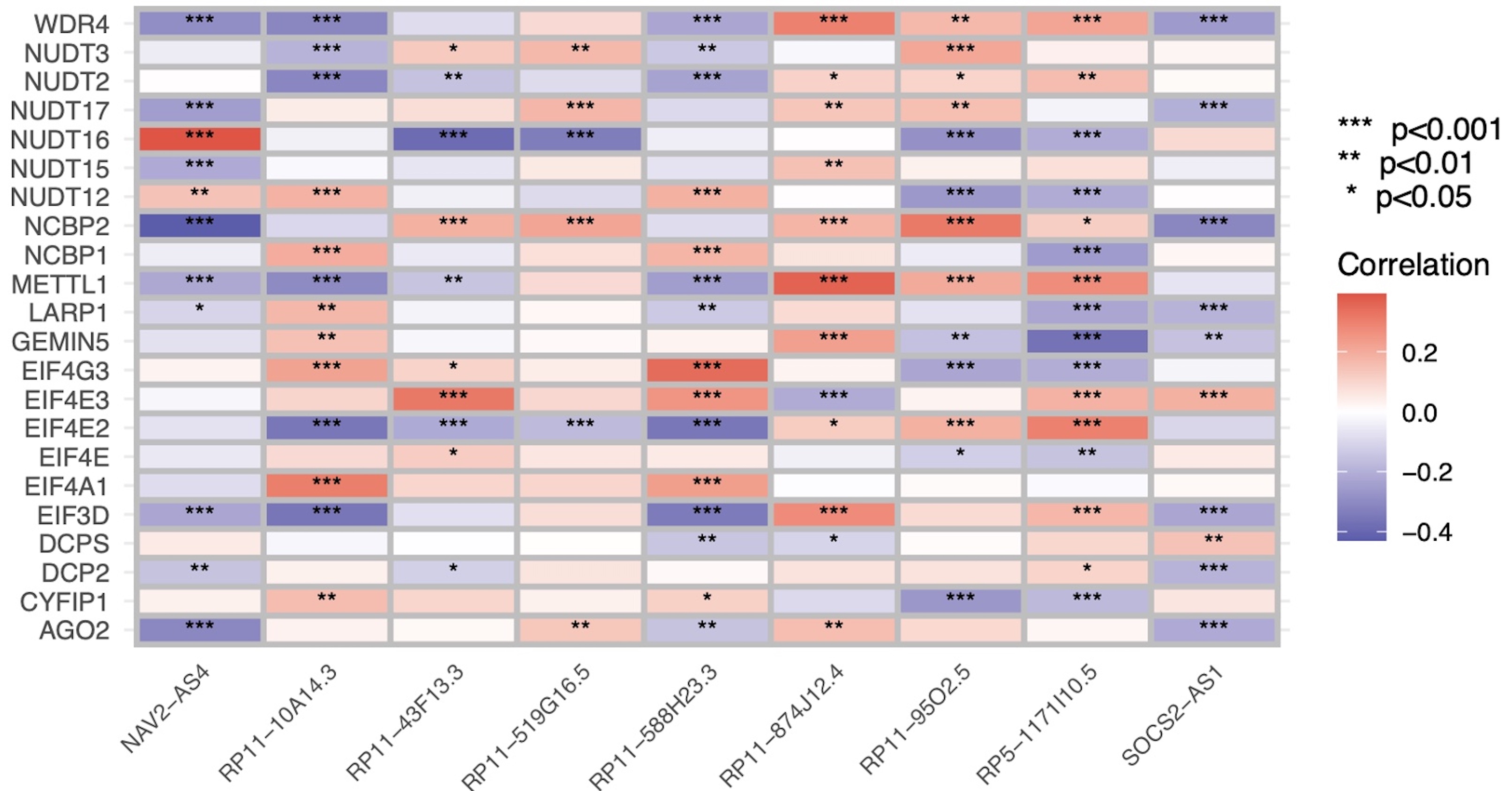

Supplement: Supplementary file 2 [file Image1.JPEG]

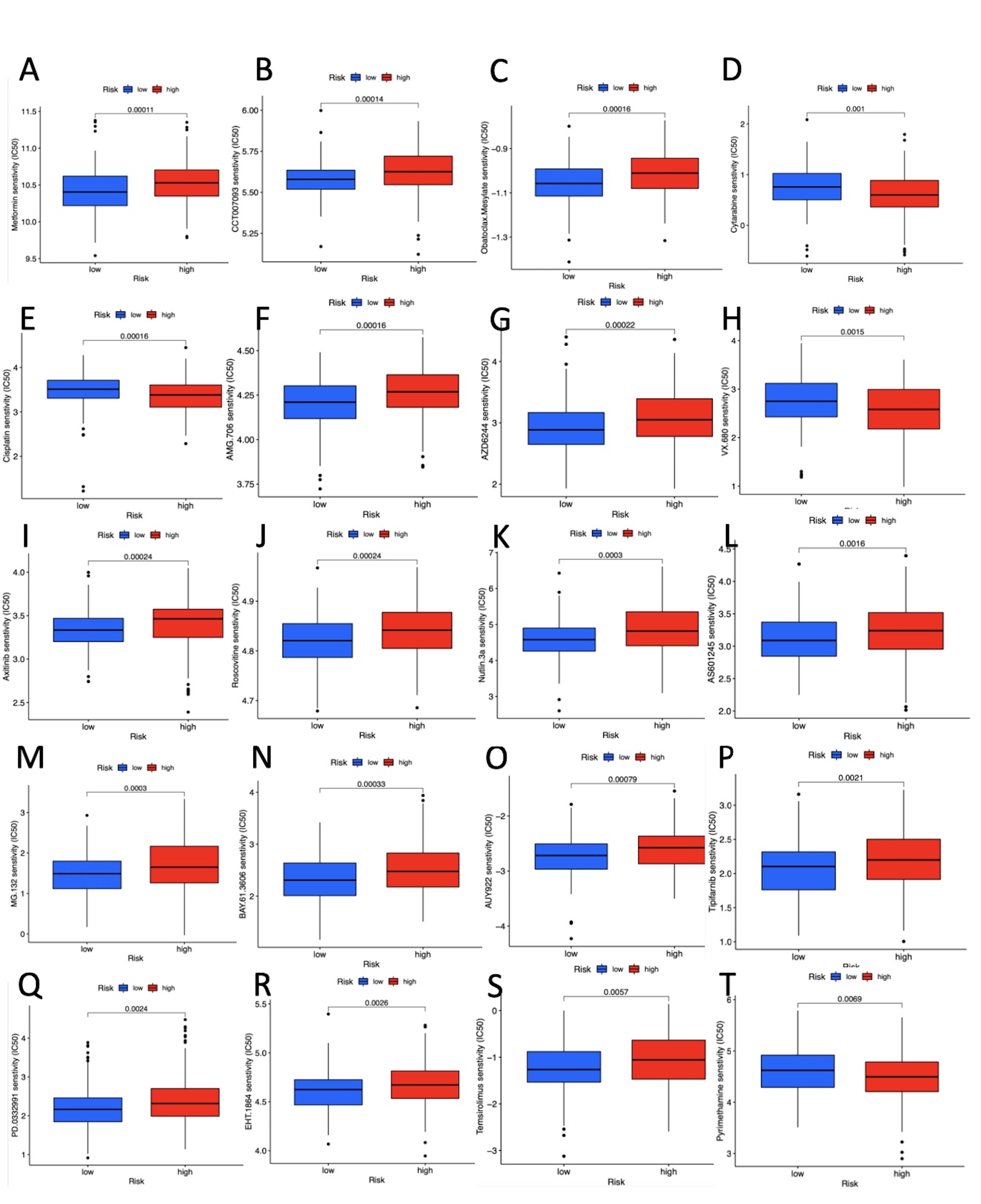

Supplement: Supplementary file 3 [file Image4.JPEG]

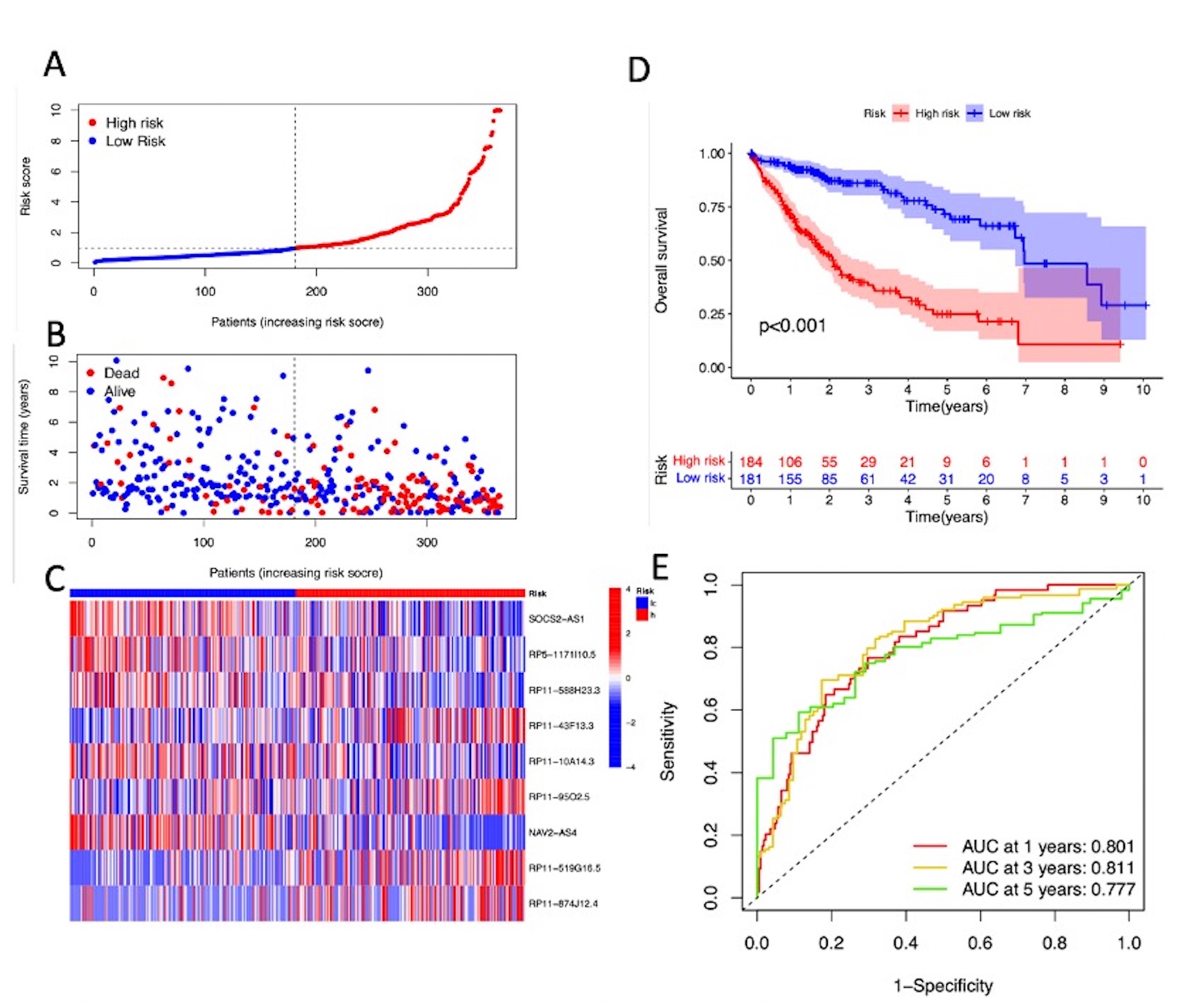

Supplement: Supplementary file 4 [file Image2.JPEG]
