# Supplementary material for: Prognostic Signature and Tumor Immune Landscape of N7-Methylguanosine-Related lncRNAs in Hepatocellular Carcinoma
Source: Front Genet. 2022 Jul 22;13:906496. doi: 10.3389/fgene.2022.906496 (PMC9354608; doi:10.3389/fgene.2022.906496)
Supplement: Supplementary file 5 [file DataSheet1.docx]

Supplementary Material

**Supplementary Table 1**. Clinical characteristic of the entire LIHC set, training set and testing set in TCGA database

| Characteristics | Type | Entire TCGA set (n=365) | Training set (n=219) | Testing set (n=146) | Pvalue |
| --- | --- | --- | --- | --- | --- |
| age | <=65 | 227 | 131 | 96 | 0.3004 |
|  | >65 | 138 | 88 | 50 |  |
| gender | female | 119 | 63 | 56 | 0.0718 |
|  | male | 246 | 156 | 90 |  |
| race | non-white | 173 | 103 | 70 | 1 |
|  | white | 182 | 109 | 73 |  |
| grade | G1-2 | 230 | 143 | 87 | 0.2503 |
|  | G3-4 | 130 | 72 | 58 |  |
|  | unknow | 5 | 4 | 1 |  |
| T | T1-2 | 271 | 166 | 105 | 0.3482 |
|  | T3-4 | 91 | 50 | 41 |  |
|  | unknow | 3 | 3 | 0 |  |
| N | N0 | 248 | 147 | 101 | 0.9027 |
|  | N1 | 4 | 3 | 1 |  |
|  | unknow | 113 | 69 | 44 |  |
| M | M0 | 263 | 158 | 105 | 0.1296 |
|  | M1 | 3 | 0 | 3 |  |
|  | unknow | 99 | 61 | 38 |  |
| stage | StageI-II | 254 | 157 | 97 | 0.2493 |
|  | StageIII-IV | 87 | 47 | 40 |  |
|  | unknow | 24 | 15 | 9 |  |


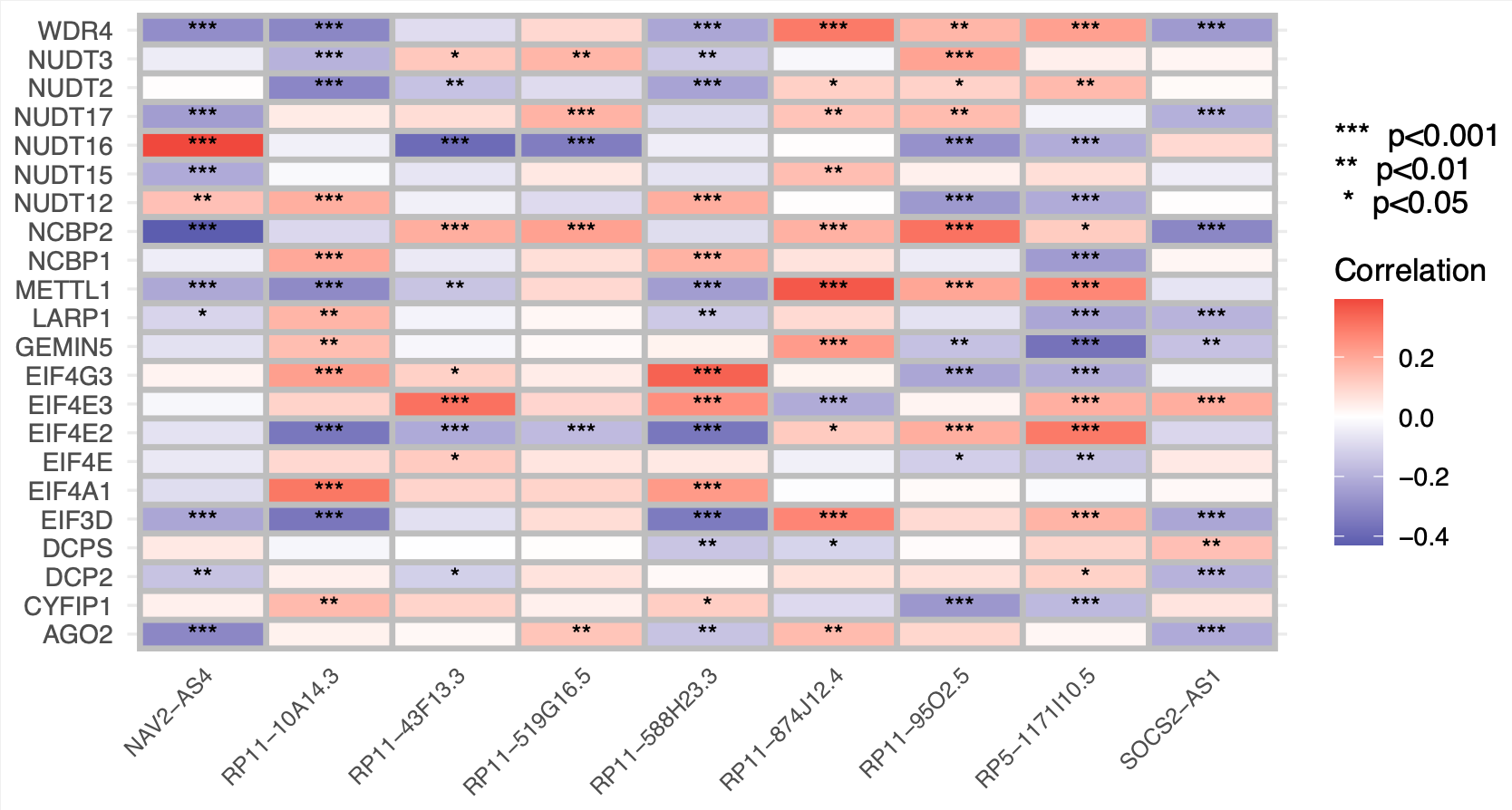


**Supplementary Figure 1**. Heatmap for the correlations between 22 m7G genes and the 9 prognostic

m7G-related lncRNAs.


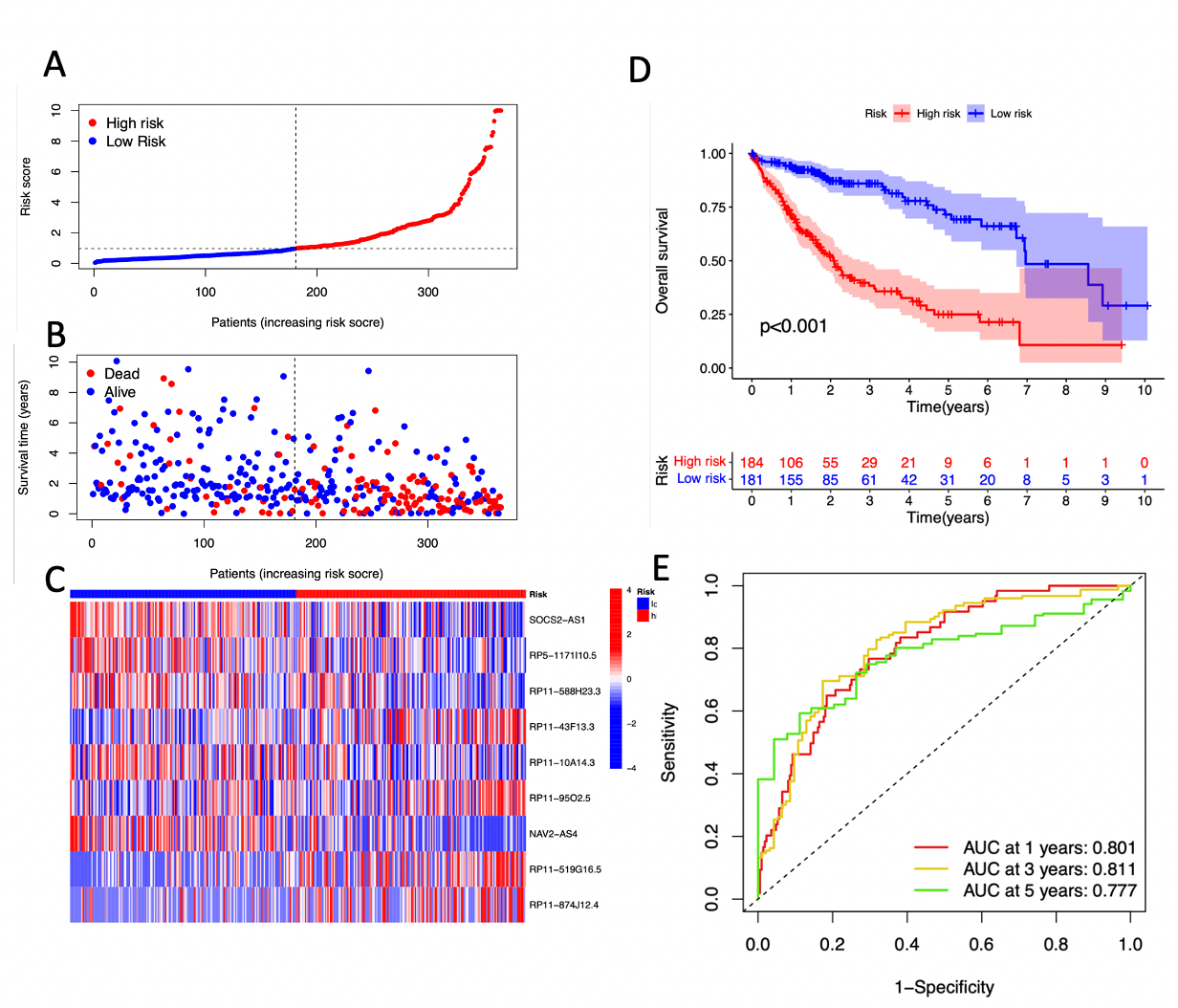


**Supplementary Figure 2**. Prognostic value of the 9 m7G-related lncRNAs risk model between the high- and low-risk groups in the entire set. (A) Distribution of risk score based on the m7G-related lncRNA model. (B) Patterns of the relationship between survival time and risk score. (C) Clustering heatmap showed the expression profile of the 9 m7G-related lncRNAs. (D) Kaplan-Meier survival curves displayed the OS of LIHC patient between high- and low-risk groups. (E) ROC curves of the risk model of 1-year, 3-year and 5-year for OS.


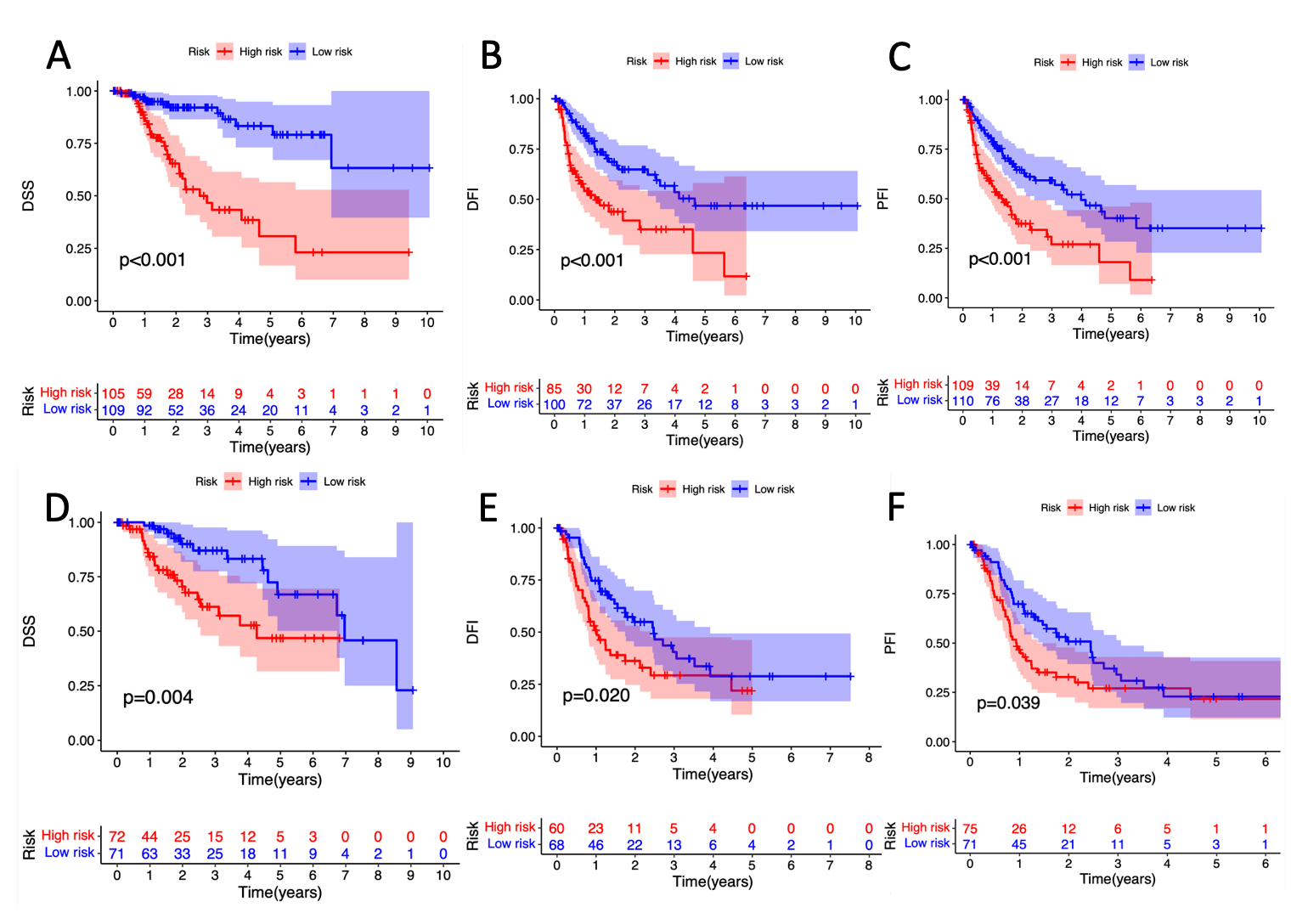


**Supplementary Figure 3.** Kaplan-Meier survival curves of the relative DSS, PFI, and DFI between the high- and low-risk groups in training and testing set. (A-C) Kaplan-Meier survival curves of the relative DSS, PFI, and DFI in training set. (D-F) Kaplan-Meier survival curves of the relative DSS, PFI, and DFI in testing set.


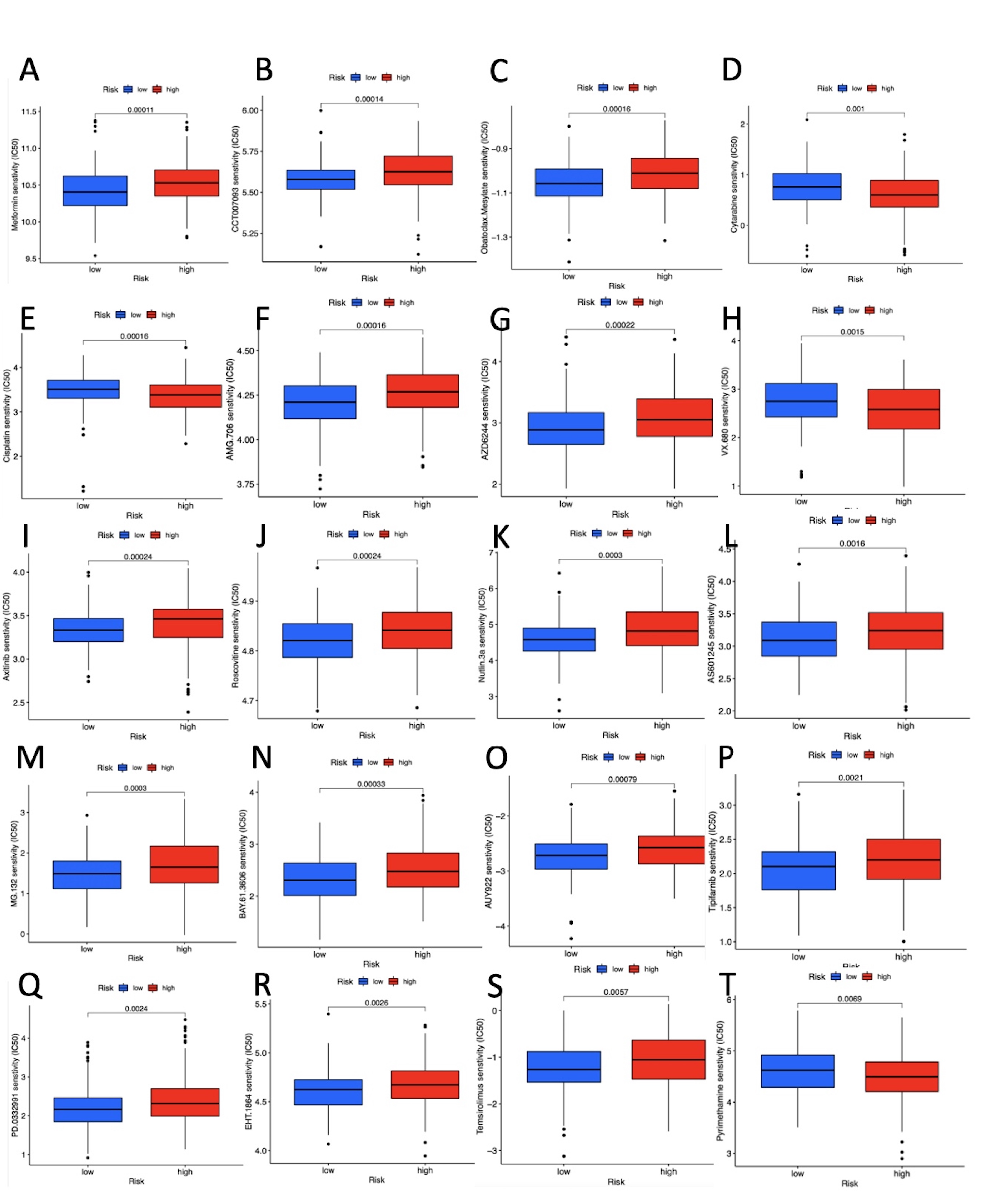


**Supplementary Figure 4**. (A-T) Identification of novel candidate compounds targeting the m7G-related lncRNA model. The model showed high risk scores were associated with a lower IC50 for chemotherapeutics.
